# Vitamin K_2_ Therapy for Postmenopausal Osteoporosis

**DOI:** 10.3390/nu6051971

**Published:** 2014-05-16

**Authors:** Jun Iwamoto

**Affiliations:** Institute for Integrated Sports Medicine, Keio University School of Medicine, 35 Shinanomachi, Shinjuku-ku, Tokyo 160-8582, Japan; E-Mail: jiwamoto@a8.keio.jp; Tel.: +81-3-3353-1211; Fax: +81-3-3352-9467

**Keywords:** vitamin K_2_, postmenopausal women, undercarboxylated osteocalcin, femoral neck, bone mineral density (BMD)

## Abstract

Vitamin K may play an important role in the prevention of fractures in postmenopausal women with osteoporosis. Menatetrenone is the brand name of a synthetic vitamin K_2_ that is chemically identical to menaquinone-4. The present review study aimed to clarify the effect of menatetrenone on the skeleton in postmenopausal women with osteoporosis, by reviewing the results of randomized controlled trials (RCTs) in the literature. RCTs that investigated the effect of menatetrenone on bone mineral density (BMD), measured by dual-energy X-ray absorptiometry and fracture incidence in postmenopausal women with osteoporosis, were identified by a PubMed search for literature published in English. Eight studies met the criteria for RCTs. Small RCTs showed that menatetrenone monotherapy decreased serum undercarboxylated osteocalcin (ucOC) concentrations, modestly increased lumbar spine BMD, and reduced the incidence of fractures (mainly vertebral fracture), and that combined alendronate and menatetrenone therapy enhanced the decrease in serum ucOC concentrations and further increased femoral neck BMD. This review of the literature revealed positive evidence for the effects of menatetrenone monotherapy on fracture incidence in postmenopausal women with osteoporosis. Further studies are required to clarify the efficacy of menatetrenone in combination with bisphosphonates against fractures in postmenopausal women with osteoporosis.

## 1. Introduction

**Figure 1:**
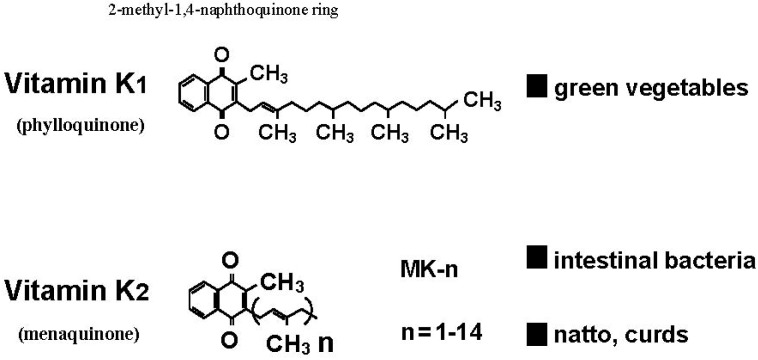
Naturally occurring forms of vitamin K—Phylloquinone (vitamin K_1_) and menaquinones (vitamin K_2_).

There are two types of naturally occurring forms of vitamin K, phylloquinone and menaquinones. All forms of vitamin K contain a 2-methyl-1,4-naphthoquinone ring as part of their structure, and individual forms differ in the length and degree of saturation of a variable aliphatic side chain attached at the 3-position (Figure 1). Phylloquinone (vitamin K_1_) is the major type of dietary vitamin K, while menaquinone-4 (vitamin K_2_) is the major form of vitamin K in the tissues, including bone. Vitamin K_1_ is supplied by the diet, especially in green leafy vegetables, while vitamin K_2_ is synthesized by bacteria in the gut. A number of foods also contain vitamin K_2_, notably *natto* (fermented soy beans), cheese, and curds, with *natto* being the richest source of vitamin K known.

**Figure 2:**
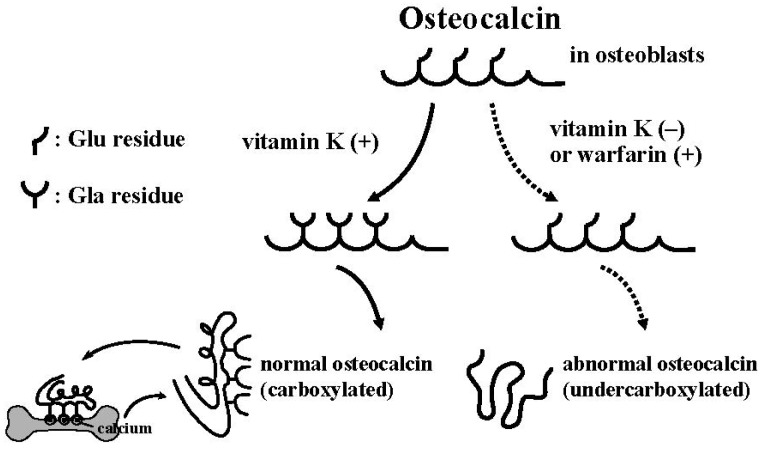
γ-Carboxylation of osteocalcin by vitamin K.

Vitamin K is known to be a cofactor of γ-carboxylase, which converts three glutamic acid (Glu) residues in osteocalcin (OC) to γ-carboxyglutamic acid (Gla), and is thus essential for γ-carboxylation of OC [1,2,3,4]. Without this modification, OC becomes undercarboxylated OC (ucOC), which lacks structural integrity and the ability to bind to the mineral hydroxyapatite (Figure 2). The carboxylation reaction is completed as an intracellular posttranslational event, and secreted OC cannot longer be carboxylated. Impaired vitamin K nutritional status or warfarin use results in high concentrations of serum ucOC, resulting in an increased risk for fractures. In Japan, the cut-off values of serum ucOC concentrations for an increased risk for fractures associated are set at 4.5 ng/mL for treatment-naïve postmenopausal women and 2.6 ng/mL for postmenopausal women treated with amino-bisphosphonates [5,6]. Thus, vitamin K may play an important role in the prevention of fractures in postmenopausal women with osteoporosis.

Menatetrenone is the brand name of a synthetic vitamin K_2_ that is chemically identical to menaquinone-4. It is approved as an anti-osteoporotic medicine by the Ministry of Health, Labour, and Welfare in Japan. Menatetrenone is therefore a drug, rather than just a simple dietary supplement, and is used as an anti-osteoporotic medicine in Asia. The possible side effects are gastrointestinal tract symptoms such as discomfort of stomach and diarrhea, because vitamin K is a fat-soluble vitamin. The contraindication of menatetrenone is warfarin use, because vitamin K set off the anticoagulant effect of warfarin. Apart from this contraindication, menatetrenone (vitamin K_2_) does not cause any serious side effects regardless of its dose [7].

A dose-finding study of menatetrenone in Japan [7] administered daily doses of 15, 45, 90, and 135 mg and revealed that 45 mg was the minimum effective dose for improving bone mass parameters evaluated by microdensitometry and/or single photon absorptiometry in postmenopausal women with osteoporosis. This optimal dose (45 mg/day) for the treatment of osteoporosis is about 150–180 times greater than the recommended daily dietary intake of vitamin K (250–300 μg) [8]. No toxic effects of menatetrenone (45 mg/day) have been reported [7]. High-dose vitamin K is needed to prevent fractures in postmenopausal women with osteoporosis [9]. However, the effect of menatetrenone on the skeleton remains a matter of controversy [10,11,12,13,14,15,16,17], and the role of menatetrenone in the treatment of osteoporosis therefore needs to be clarified. In clinical practice, menatetrenone is frequently used in combination with bisphosphonates.

The objectives of the present study was to clarify the effects of menatetrenone on serum ucOC concentrations, bone mineral density (BMD), and fracture incidence in postmenopausal women with osteoporosis, by reviewing the results of randomized controlled trials (RCTs) in the literature.

## 2. Approaches to Determining the Effect of Menatetrenone on Bone Health

RCTs that investigated the effect of menatetrenone on the skeleton of postmenopausal women with osteoporosis were identifiedby a PubMed search for literature published in English using combinations of terms “vitamin K_2_ or menatetrenone”, “bone”, “postmenopausal women”, and “osteoporosis”. The effects of menatetrenone on the skeleton were analyzed based on the data from the RCTs retrieved. The endpoints included BMD measured by dual-energy X-ray absorptiometry and fracture incidence. Eight studies met the criteria for RCTs on the effect of menatetrenone on the skeleton of postmenopausal women with osteoporosis [10,11,12,13,14,15,16,17]. Of eight RCTs, one included postmenopausal women with osteopenia or osteoporosis [15].

## 3. Menatetrenone Monotherapy

Menatetrenone reduces serum ucOC concentrations [17,18]. The anti-fracture efficacy of menatetreone in postmenopausal osteoporosis has been shown in eight RCTs (Table 1) [10,11,12,13,14,15,16,17]. Of the eight RCTs, six were performed in Japan, one in Indonesia, and one in China. One Japanese RCT was a phase IV trial (Osteoporotic Fracture (OF) study) conducted in a large sample of postmenopausal women with osteoporosis (*n* = 4378) [13]. The dose of menatetrenone was 45 mg/day in all these studies. Placebo controls were used in one RCT, non-treatment controls in four RCTs, calcium supplementation controls in two RCTs, and active controls (alfacalcidol) in one RCT. The number of study subjects ranged from 44–4378 in the menatetrenone and control groups, and the study periods ranged from 1–3 years.

Except for the OF study, RCTs with small sample sizes showed non-significant or modest effects on BMD of the lumbar spine and distal radius, and similarly low efficacy against fractures (mainly vertebral fractures) in postmenopausal women with osteoporosis (Table 1). A pivotal Japanese RCT conducted by Shiraki *et al* [10], showed that menatetrenone modestly increased lumbar spine BMD and reduced the incidence of clinical fractures in postmenopausal women with osteoporosis (relative risk: 0.45). A post-hoc analysis of the OF study showed a decrease in the incidence of vertebral fractures in postmenopausal women with a history of at least five vertebral fractures (relative risk: 0.61). There was an absence of a significant anti-fracture effect of menatetrenone in the subjects as a whole in the OF study [13]. The reason for this result was that many of the patients enrolled in the study might have had mild osteoporosis and a lower risk of developing fractures, probably because of the use of less specific diagnostic criteria for osteoporosis, which were only based on radiographic grading of bone atrophy and disregarded other risk factors for fractures [13].

## 4. Combined Menatetrenone and Alendronate Therapy

Hirao *et al.* [19] conducted a RCT to examine the benefit of combined menatetrenone and alendronate therapy in postmenopausal women with osteoporosis. The increase in femoral neck BMD and the decrease in serum ucOC concentrations were greater in the alendronate plus menatetrenone group compared with the alendronate alone group.

## 5. Discussion

Impaired vitamin K nutritional status impairs γ-carboxylation of OC (vitamin K-dependent protein). Although OC is a calcium-binding protein that is strongly expressed in bone, the effects of OC on bone remain obscure. However, a biomechanical analysis of the quality of the femora in OC-deficient mice showed that the yield energy (ductility of bone specimens), failure load (strength of the bone), and stiffness (elasticity of the bone) decreased dramatically after ovariectomy [20], suggesting that OC may have an important role in bone biology, and that OC deficiency may be associated with bone fragility. Thus, vitamin K may play an important role in maintaining bone health.

**Table 1:** Effects of menatetrenone on bone mineral density (BMD) and fracture incidence in postmenopausal women with osteoporosis: Randomized controlled trials.

	Authors	Reference (year)	Groups (number of subjects)	Study design	Outcome of menatetrenone
BMD	Shiraki *et al.*	[10] (2000)	Menatetrenone (120), non-treatment (121)	Open-label RCT in Japan (45 mg, 2 years)	Lumbar spine BMD loss mitigated Menatetrenone: −0.5%, non-treatment: −3.3%
	Iwamoto *et al.*	[14] (2000)	Menatetrenone (22), alfacalcidol (29), combination (21), calcium (22)	Open-label RCT in Japan (45 mg, 2 years)	Lumbar spine BMD maintained Menatetrenone: +0.9%, calcium: −0.79%
	Iwamoto *et al.*	[11] (2001)	Menatetrenone (23), etidronate (25), calcium (24)	Open-label RCT in Japan (45 mg, 2 years)	Ultra distal radius BMD maintained Menatetrenone: −0.1%, calcium: −1.7%
	Ushiroyama *et al.* *	[15] (2002)	Menatetrenone (43), alfacalcidol (43), combination (43), non-treatment (43) (Subjects: Postmenopausal women with osteopenia or osteoporosis)	Open-label RCT in Japan (45 mg, 2 years)	Lumbar spine BMD increased Menatetrenone: +1.37%, non-treatment: −4.05%
	Ishida & Kawai	[13] (2004)	Menatetrenone (66), estradiol (66), etidronate (66), eel calcitonin (66) alfacalcidol (66), non-treatment (66)	Open-label RCT in Japan (45 mg, 2 years)	Distal radius BMD loss mitigated Menatetrenone: −1.9%, non-treatment: −3.3%
	Purwosunu *et al.*	[16] (2006)	Menatetrenone (33), placebo (30)	Double-blind RCT in Indonesia (45 mg, 1 year)	Lumbar spine BMD increased Menatetrenone: +1.74%, placebo: −0.18%
	Jiang *et al.*	[17] (2014)	Menatetrenone (118), alfacalcidol (118)	Double-blind RCT in China (45 mg, 1 year)	Lumbar spine BMD increased Menatetrenone: 1.2%, alfacalcidol: 2.2%
Fracture	Shiraki *et al.*	[10] (2000)	Menatetrenone (120), non-treatment (121)	Open-label RCT in Japan (45 mg, 2 years)	Clinical fracture (mainly vertebral fracture) incidence decreased Menatetrenone: 10.9%, non-treatment: 30.3%
	Iwamoto *et al.*	[11] (2001)	Menatetrenone (23), etidronate (25), calcium (24)	Open-label RCT in Japan (45 mg, 2 years)	Vertebral fracture incidence decreased Menatetrenone: 8.7%, calcium: 25%
	Ishida & Kawai	[12] (2004)	Menatetrenone (66), estradiol (66), etidronate (66), eel calcitonin (66), alfacalcidol (66), non-treatment (66)	Open-label RCT in Japan (45 mg, 2 years)	Vertebral fracture incidence decreased Menatetrenone: 14%, non-treatment: 26%
	Inoue *et al.*	[13] (2009)	Menatetrenone (2193), non-treatment (2185)	Open-label RCT in Japan (45 mg, 3 years) (Osteoporotic Fracture study)	No significant effect on vertebral fracture incidence Menatetrenone: 5.87/100 patient-years Non-treatment: 5.74/100 patient-years

* Study subjects included were postmenopausal women with osteopenia or osteoporosis. BMD: Bone mineral density; RCT: randomized controlled trial.

The present review revealed positive evidence for the effect of menatetrenone monotherapy on BMD and the incidence of fractures (mainly vertebral fractures) in postmenopausal women with osteoporosis. However, this evidence was derived from RCTs with small sample sizes. Thus, menatetrenone is used as a second-line medicine for the treatment of postmenopausal osteoporosis in Japan [8]. Knapen *et al.* [21] reported that menatetrenone (45 mg/day) improved hip bone geometry, bone strength indices, and bone mineral content in healthy postmenopausal women, supporting the effect of menatetrenone on bone health.

The mechanism whereby menatetrenone exerts its protective effect against fractures remains uncertain. Menatetrenone reduced the incidence of clinical fractures despite causing no significant change or only a modest increase in BMD. However, menatetrenone is capable of improving bone quality (material property) and preventing fractures. As noted above, impaired vitamin K nutritional status results in impaired γ-carboxylation of OC, and one possible explanation for its protective effect against fractures is that menatetrenone may promote γ-carboxylation of OC and induce the production and secretion of OC by osteoblasts. However, the vitamin K intake (500 μg/day) needed for full γ-carboxylation of OC may be approximately 100-fold lower than the dose of menatetrenone (45 mg/day) needed to produce a clinical effect [9].

The present review investigated the effect of an anti-osteoporotic medicine, menatetrenone (vitamin K_2_) on the skeleton in postmenopausal women with osteoporosis. However, vitamin K_1_ is also considered to contribute to bone health, because all vitamin K molecules are converted into menaquinone-4 (vitamin K_2_) [22] and then become active. So, the effects of vitamin K_1_ on bone parameters appear to be equivalent to those of vitamin K_2_. Cheung *et al.* [23] reported that vitamin K_1_ (5 mg/day) did not protect against age-related decline in BMD, but prevented clinical fractures in postmenopausal women with osteopenia. The World Health Organization has proposed a set of guidelines for the diagnosis of osteoporosis in adult women based on a measurement of BMD expressed as the number of SD below young adult mean (t-score); [24] osteopenia (low bone mass) is defined as BMD between 1 and 2.5 standard deviation (SD) below the mean value of peak bone mass in young normal women, while osteoporosis is defined as BMD more than 2.5 SD below the mean value of peak bone mass in young normal women [24]. The results of Cheung study appeared to be consistent with those of menatetrenone.

A small RCT showed the beneficial effects of combined menatetrenone and alendronate therapy on serum ucOC concentrations and femoral neck BMD in postmenopausal women with osteoporosis [19]. This result suggests the possibility that menatetrenone could serve as an adjuvant of bisphosphonates in the treatment of postmenopausal osteoporosis. However, it remains uncertain whether this combination therapy is more useful for preventing fractures than single therapy with bisphosphonates. Thus, menatetrenone appears to be a second-line medicine in postmenopausal women with osteoporosis [8].

The present review showed the effects of menatetrenone on serum ucOC concentrations, BMD, and fracture incidence in postmenopausal women with osteoporosis. The study subjects of eight RCT analyses did not always have higher serum ucOC concentrations. Therefore, it is not established whether menatetrenone is more effective for reducing fractures in postmenopausal osteoporotic women with higher serum ucOC concentration than those with lower serum ucOC concentrations, despite the fact that high serum ucOC concentrations are related to an increased risk for fractures in postmenopausal women with osteoporosis. Further studies are needed to clarify this issue.

## 6. Conclusions

The present review revealed positive evidence for the effects of menatetrenone monotherapy on fracture incidence in postmenopausal women with osteoporosis. However, this evidence was derived from RCTs with small sample sizes. The efficacy of combined bisphosphonate and menatetrenone therapy against fractures compared with bisphosphonate monotherapy remains to be established. Currently, menatetrenone is a second-line medicine for the treatment of postmenopausal osteoporosis.
